# The Impact of the SARS-COVID-19 Lockdowns on the Subjectively Perceived Performance Level of Amateur Athletes after Returning to the Gyms

**DOI:** 10.3390/jfmk9020059

**Published:** 2024-03-27

**Authors:** Maria A. Bernstorff, Norman Schumann, Charlotte Cibura, Julius Gerstmeyer, Thomas A. Schildhauer, Matthias Königshausen

**Affiliations:** 1 Universitätsklinik Bergmannsheil Bochum, Medical Department of Ruhr University of Bochum, 44789 Bochum, Germany; charlotte.cibura@bergmannsheil.de (C.C.); julius.gerstmeyer@bergmannsheil.de (J.G.); thomas.schildhauer@bergmannsheil.de (T.A.S.); matthias.konigshausen@gmail.com (M.K.); 2 Institution for Mathematics, Ruhr University Bochum, 44801 Bochum, Germany; norman.schumann@ruhr-universitat-bochum.de

**Keywords:** SARS-COVID-19, lockdown, fitness, amateur sports, prevention

## Abstract

Since December 2019, few issues have garnered as much global attention as severe acute respiratory syndrome coronavirus 2 (SARS-CoV-19). The imposed lockdowns in 2020/21, which led to the closure of all gyms, barred people from participating in their favourite sports activities. This study explores athletes’ self-evaluations of their performance levels upon return to training facilities post-reopening. Data were collected in May 2021, after the end of the second lockdown, using a national online questionnaire. The study recorded 20 demographic and training-specific parameters to discern the factors influencing self-perceived performance upon resuming gym activities after the 2020/2021 lockdown. A total of 1378 respondents participated in the study. Of the total number of participants, 27.5% (365) reported regaining 100% of their original performance level after reopening their studios, a proportion that comprised 212 males, 150 females, and 3 individuals of unspecified gender. Additionally, 35.7% (474) estimated their performance level to be up to 75%, followed by 30% (398) recording their performance level at 50%, and a minority of 6.8% (90) determining their performance level to be up to 25%. Exercise intensity prior to lockdown, training experience, sex, and concurrent practice of another sport significantly influenced the athletes’ self-assessment of their current fitness levels (*p* > 0.001, *p* > 0.001, *p* > 0.001, and 0.006, respectively). We need to understand the factors that shape self-perception, especially in case of another lockdown, in order to provide preventive assistance concerning mental and physical well-being. Positive influences on self-perception include prior athletic experience, intensive training before the lockdown, and continued participation in sports throughout the lockdown. Younger age is also favourable, but this may not necessarily reflect the benefits of youth; rather, it could indicate the current lack of accessible online sports activities for older individuals. Women, however, might have a less favourable perception of their own athletic performance.

## 1. Introduction

Since December 2019, few issues have consumed the world as thoroughly as severe acute respiratory syndrome coronavirus 2 (SARS-COVID-19). The disease itself, coupled with the societal impact of recurrent lockdowns and social distancing, has profoundly affected people’s mental and physical health. For many, sport is a fundamental aspect of life, acting as a vital link between body and mind. It not only helps sustain physical and mental well-being but also reinforces socio-communal bonds [1,2,3,4,5,6].

In recent years, there has been a significant increase in ambitious amateur athletes engaged in fitness and weight training. Many of these enthusiasts train at a level comparable to professional athletes. A survey conducted by a German Health insurance company indicated that 36% of the population claimed to work out in a gym.

However, a subsequent study by Deloitte revealed that the SARS-COVID-19 pandemic and the resulting closure of gyms led to a slowdown in the annual membership growth rate. Despite this setback, general fitness remains the top choice among organized sports in Germany, with 9.26 million registered members.

In the years during and after the SARS-COVID-19 pandemic, many effects on physically active people were studied. Over the years both the long- and the short-term effects were studied [7,8,9]. Professional athletes and organized club sports in particular allowed systematic studies to be carried out [2,10,11,12,13,14]. Popular fitness sports as a whole are often difficult to capture due to their heterogeneity because frequently, individuals undertake these sports on their own, often without any connection to a trainer and at very different intensities. Nevertheless, these sports are some of the most frequently practiced sports and are therefore extremely important.

The lockdowns in 2020 and 2021, during which all gyms were closed, meant that many Germans were unable to participate in their favourite recreational sport. For many, the closure of gyms not only resulted in the loss of a favoured pastime but also eliminated a significant source of physical activity.

This study explores athletes’ self-evaluation of their performance levels after returning to fitness studios following a lockdown. Using this self-assessment data, we aim to effectively identify factors that can aid in the management of future facility closures. The aim was to determine what subjective fitness level the respondents felt they were at when they returned to the studios and which factors led to a better or worse self-evaluation. 

A subjectively negative perceived performance level can go hand in hand with lower performance. Nevertheless, amateur athletes often start at a similar performance level to where they left off a long time ago. As many studies have shown, this can lead to a higher loss of performance and injury rates [13,14,15,16,17].

## 2. Materials and Methods

Data were gathered from a nationwide online questionnaire distributed in May 2021 after Germany came out of its second lockdown. The Ruhr University Bochum’s ethics committee, under the ethics committee number 18-6729 BR, approved this study. There was also a review of the data protection regulations by Ruhr University’s data protection officer. Various institutions and media outlets that are focused on health and fitness helped promote the survey online.

In the run up to the study we performed a statistical analysis to identify a representative study group. The survey was conducted online and anonymously. All respondents were presented with the privacy policy and voluntarily agreed to participate in the study. Individuals of all genders and ages had the opportunity to take part in this survey. Informed consent was obtained from all participants or their legal guardians as necessary. The questionnaire was disseminated through various channels, including social media platforms. Prominent gym chains, such as FitX^®^ (a leading gym chain in Germany), shared the survey link with their members, newspaper articles were published to raise awareness, and multiple physical therapy and medical associations actively participated in distributing the survey. Furthermore, support for this study was provided by organisations such as the Functional Fitness Association, CrossFit^®^ Boxes, and the Bodybuilding Association Germany^®^.

We collected 12 demographic or training-specific parameters with which to identify the factors influencing athletes’ perceived performance levels upon returning to the gym after the 2020/2021 lockdown from December 2020 to May 2021. We recorded factors such as age, gender, weight, height, training experience, previous training intensity and the way in which they planned their training. The self-assessment question focused on the athlete’s subjective perception of their performance level compared with their pre-lockdown level upon their return to the gym after two lockdowns. The additional collected information regarding the individual and their training habits was used to help identify the defining factors that allow athletes to believe they have a high subjective performance level when they return to the gym. These questions are provided in Table 1 and were translated into English for this paper, as the original questionnaire was disseminated in German.

### Statistical Analysis

Calculations and visualizations were conducted using Python 3.8 (Python Software Foundation. Python Language Reference, version 2.8), Jupyter 1.0.0 (Kluyver, T. et al., 2016. Jupyter Notebooks), and packages including pandas 1.2.0, numpy 1.19.4, seaborn 0.11.1, and scipy 1.5.4. The chi-square test (Pearson or Fisher, contingent on sample size) was persistently applied to examine stochastic independence in the contingency tables. We always reported the *p*-value with the null hypothesis; for example, “H0: Characteristics X and Y are stochastically independent” was significantly rejected at an alpha level of 0.05. The statistical characteristics X and Y in this hypothesis are discussed in the text.

## 3. Results

The study included a total of 1378 participants. Data collected encompassed both personal and training-specific factors. Additionally, participants were asked to list any other physical activities they engaged in, like physically demanding jobs or other sports, beyond their gym workouts.

Of the 1378 participants, 719 (52.17%) were female, 663 (48.11%) were male, and 5 (0.36%) identified as diverse. There were no specified age restrictions for this group. Participant’s height and weight were recorded to determine their physical composition. The calculated body mass index (BMI) for each participant is presented in Table 2.

Of the participants, 207 (15.02%) reported having 1–2 years of experience working out in a gym, followed by 194 (14.08%) with 2–3 years, 506 (36.72%) with 3–7 years, and 480 (34.83%) with 8 or more years. The majority went to the gym 3–4 times per week.

When considering exercise duration, 558 (40.49%) participants exercised up to 5 h per week, 620 (44.99%) between 5 and 10 h weekly, 171 (12.41%) up to 15 h, and only 35 (2.54%) participants reported exercising over 15 h weekly.

For weekly frequency, 537 (38.97%) exercised 3–4 times, 413 (29.97%) 1–2 times, 360 (26.12%) 4–6 times, and 77 (5.59%) exercised daily.

Altogether, 925 (67.13%) participants followed a training plan. Of these, 477 (34.62%) created their own plan, 406 (29.46%) had a trainer prepare it for them, and 42 (3.05%) followed an online plan. Exercise durations ranged from 1 to 35 h per week, averaging 4 h per week (Table 3).

The physical activity recorded during work showed that 711 individuals (51.6%) had a physically inactive job. Meanwhile, 298 (21.63%) stated they were moderately active (2–4 h daily), 287 (20.83%) reported being actively engaged in their job (4–8 h), and 91 (6.6%) identified as being highly active (>8 h) (Table 4). When inquired about other sports activities apart from fitness training, 732 (53.12%) responded affirmatively. Of these, 634 (46.01%) participated recreationally and 101 (7.33%) competitively. The most common activities were running, cycling, and team sports.

**Figure 1:**
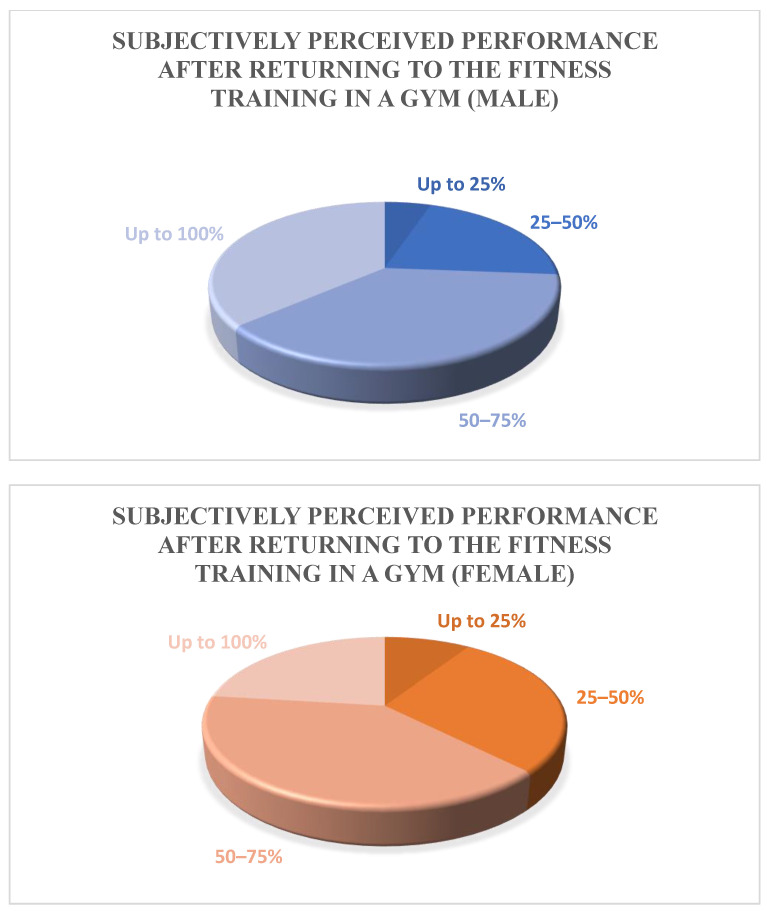
Illustrated comparison of the different assessments of the subjective fitness levels of men and women.

Out of all respondents, 27.5% (365 individuals) reported returning to their previous performance level after the reopening of the studios. This group consisted of 212 males, 150 females, and 3 unspecified genders. A further 35.7% (474 individuals) estimated their performance at up to 75% of their previous level, consisting of 218 males, 256 females, and 1 unspecified gender. Thirty percent (398 individuals) estimated their performance at only 50%, with this group consisting of 123 males and 184 females, with no respondents being of an unspecified gender. The remaining 6.8% (90 individuals) estimated their performance at a mere 25% of their previous level, this proportion consisted of 30 males, 60 females and no respondents of an unspecified gender (Table 5, Figure 1).

Variables significantly influencing the individuals’ subjective fitness levels included the intensity of pre-lockdown training (frequency of training sessions per week, *p* > 0.005), number of years of training experience (*p* > 0), sex (*p* > 0), and concurrent participation in another sport (*p* = 0.006). Notably, a focus on running was a significant factor (*p* = 0.006).

## 4. Discussion

This study focused on athletes’ self-evaluation of their fitness levels upon returning to gyms in 2021 after the closures imposed due to the SARS-COVID-19 pandemic. The survey involved recreational athletes who regularly exercised in a gym. To our knowledge, there are no other studies in the literature that attempt to look at the very heterogeneous sport of “fitness” as a whole. We recorded their self-perceived current performance levels relative to their pre-pandemic levels. 

Upon reopening, 27.5% of all participants (212 males, 150 females, and 3 others) reported that they had regained 100% of their prior performance. A proportion of 35.7% (218 males, 256 females, 1 other) believed they were operating at up to 75%, 30% (123 males, 184 females) estimated themselves to be at 50%, and 6.8% (30 males, 60 females) felt they were functioning at up to 25%.

The data reveal that less than one-third of participants were not operating at their full performance capacity. Looking at these data against the background of current literature, one can assume a correlation between subjectively perceived performance and objective performance. Some studies, especially in competitive or team sports, have found that there were higher injury rates after returning to sport after the SARS-COVID-19 break [14,15,17]. It can therefore be assumed that the number of injuries in recreational fitness sports may also have increased. However, as a limiting factor the study focused on the athletes’ self-assessments, not necessarily their actual performance levels.

The data reveal a significant effect of training intensity (weekly training units or hours) and experience (in years) prior to the lockdown on an athlete’s perceived fitness level afterward. Although no explicit study on this aspect has been carried out, a comparison can be made with professional athletes, who often found returning to their sport easier post-lockdown [17,18,19,20]. This implies that having higher training intensity and more experience before lockdown leads to easier sports reintegration afterward.

Interestingly, younger athletes and those involved in individual sports often increased their sporting activities during the lockdown, thereby improving their fitness levels compared with those who were less active or inactive [2,3,8,21,22]. Other studies indicate that older athletes (over 55 years) and team sports athletes encountered more difficulties in maintaining regular sporting activities during and after the lockdown [4,19,23]. For older athletes [24], this could be due to the less accessible online resources provided by gyms or sports facilities. The absence of the social aspect of team sports during the lockdown also resulted in lower motivation to exercise and, thus, less sporting activity [19,22,23]. 

However, due to more time spent at home and flexible working hours, many people increased their physical activity [3,8]. However, our data show that only 27.5% of participants regarded their fitness level as 100% post-lockdown. It is noteworthy that female respondents were significantly more likely to rate their fitness level lower than males (male = 212, female = 150, out of a total male population of 583 and female population of 650). No significant influence of gender could be identified in the literature so far. In the present study, however, it was shown that the self-perception of performance is often lower in the female gender than in the male gender. This mirrors other studies that have evaluated gender differences in anxiety, uncertainty, and athletic performance post-lockdown, all of which have concluded that women often return to sport with more self-doubt. However, if one observes all of the literature, regardless of SARS-COVID-19, this seems quite logical; for instance, women are affected by self-doubt much more frequently than men [25]. This study is a self-report and acknowledges the role of subjective perception and self-doubt in assessing one’s fitness.

This study has several noteworthy limitations. Firstly, the cross-sectional nature of the study makes it challenging to draw definitive conclusions. Secondly, our internet-based voluntary recruitment may have introduced significant selection bias. More specifically, it may have excluded those without internet access and potentially attracted respondents specifically interested in the topic, thereby inducing self-selection bias.

During the 2020/2021 lockdown stemming from the SARS-COVID-19 pandemic, the public was urged to maintain physical activity despite the shutdown of sports facilities. This was in light of the various benefits of exercise. However, studies indicate that this goal was often not met, particularly among certain demographic groups.

## 5. Conclusions

The SARS-COVID-19 pandemic and ensuing lockdowns significantly impacted both physical fitness and mental health across the population. We focused on how recreational athletes from the general public perceived their fitness levels upon returning to gyms following the 2020/2021 lockdowns. Key factors affecting these perceptions appeared to be prior athletic experience, intensive training before lockdown, and the maintenance of regular exercise during lockdown. One positive, yet uncontrollable, factor was youth. However, this factor is presumed to relate more to the limited accessibility of online sporting activities for older individuals rather than age itself. Females tended to have a more negative self-perception of their athletic performance. The quintessence of this study is that sporting activity must be accessible to all population groups. Both the older population and children must receive motivating online or outdoor offers in order to maintain a high level of sporting activity. The accessibility of sports activities (e.g., for the older population group) as well as the fun factor (e.g., group training through internet offers for the younger population) should be guaranteed easily and free of charge. Awareness of these contributing factors could be important if future lockdowns occur, allowing for measures with which to support the mental and physical health of individuals. Regardless of the SARS-COVID-19 pandemic, this study shows that sport is an important factor for fitness and self-confidence. The more active the respondents were before the pandemic, the easier it was for them to return to sport after the pandemic.

## Figures and Tables

**Table 1:** Online distributed questionnaire (translated in English and adjusted in format).

Year of birth
Weight (in kg)
Height (in cm)
Sex
How many years have you been working out in the gym, or how many years have you been working out in the gym in the past, if you are no longer working out in the gym?
O 1–2 years
O 2–3 years
O 3–7 years
O >8 years
How often do you exercise per week (eventually additionally at home)?
O 1–2 times a week
O 3–4 times a week
O 4–6 times a week
O Daily
Do you follow a training plan (if so, who developed the plan)?
O Yes, created on my own
O Yes, created online
O Yes, created by a coach
O No
Enter the total hours of your fitness training.
Do you regularly participate in other sports besides fitness?
What other sports do you do besides fitness training and at what level (competitive, hobby)?
How many hours do you work physically?
O physically highly active (>8 h)
O physically active (4–8 h)
O physically moderate active (2–4 h)
O physically inactive (0–2 h)
At what percentage of your original performance level would you rate yourself at times when gyms opened up again after the SARS-COVID-19 pandemic 2021/2022?
O 0–25%
O 25–50%
O 50–75%
O 75–100%

**Table 2:** Distribution of the athletes’ personal data.

Personal Data	
Sex Distribution	
Male	N = 663 (48.11%)
Female	N = 719 (52.17%)
Diverse	N = 5 (0.36%)
BMI	
Underweight: ≤18.5	N = 28 (2.03%)
Normal/ideal: 18.5–25	N = 733 (53.19%)
Overweight: 25–30	N = 438 (31.79%)
Obese: 30–35	N = 120 (8.71%)
Extremely obese: ≥35	N = 58 (4.21%)

**Table 3:** Training-specific data.

Training-Specific Data	
Years of training in a gym	
1–2 years	N = 207 (15.02%)
2–3 years	N = 194 (14.08%)
3–7 years	N = 506 (36.72%)
≥8 years	N = 480 (34.83%)
Frequency of training per week	
1–2 times per week	N = 413 (29.97%)
2–3 times per week	N = 537 (38.97%)
4–6 times per week	N = 360 (26.12%)
Daily	N = 77 (5.59%)
hours of training per week	
≤5 h	N = 558 (40.49%)
5–10 h	N = 620 (44.99%)
10–15 h	N = 171 (12.41%)
>15 h	N = 35 (2.54%)

**Table 4:** Activity besides fitness training.

Activity Besides Fitness Training	
Physical activity at work	
Inactive (0–2 h)	N = 711 (51.6%)
Moderately active (2–4 h)	N = 298 (21.63%)
Active (4–8 h)	N = 287 (20.83%)
Highly active (>8 h)	N = 91 (6.6%)
Performing other sports	
Yes	N = 732 (53.12%)
No	N = 655 (46.01%)

**Table 5:** Subjectively perceived performance after returning to fitness training in a gym.

Subjectively Perceived Performance after Returning to the Fitness Training in a Gym	
Up to 25%	N = 90 (m = 30, f = 60, d = 0)
25–50%	N = 398 (m = 123, w = 184, d = 0)
50–75%	N = 474 (m = 218, f = 256, d = 1)
Up to 100%	N = 365 (m = 212, f = 150, d = 3)

## Data Availability

Data are contained within this article.
